# Anxiety and Anger Symptoms in Hwabyung Patients Improved More following 4 Weeks of the Emotional Freedom Technique Program Compared to the Progressive Muscle Relaxation Program: A Randomized Controlled Trial

**DOI:** 10.1155/2015/203612

**Published:** 2015-10-11

**Authors:** Jin Woo Suh, Sun Yong Chung, Sang Young Kim, Jung Hwan Lee, Jong Woo Kim

**Affiliations:** ^1^Sungmo-maum Oriental Clinic, 239-53 Muk-dong, Jungnang-gu, Seoul 131-852, Republic of Korea; ^2^Department of Oriental Psychiatry, Gangdong Kyung Hee University Hospital, 149 Sangil-Dong, Gangdong-gu, Seoul 134-837, Republic of Korea; ^3^Hyeminseo Oriental Clinic, 217-8 Mia-dong, Gangbuk-gu, Seoul 142-810, Republic of Korea

## Abstract

*Background*. The Emotional Freedom Technique (EFT) is a meridian-based psychological therapy. The present clinical trial investigates the effectiveness of EFT as a new treatment option for Hwabyung (HB) patients experiencing anger and compares the efficacy to the Progressive Muscle Relaxation (PMR), the conventional meditation technique.* Methods*. The EFT and progressive muscle relaxation (PMR) methods were performed on 27 HB patients, and their capacities to alleviate anxiety, anger, and emotional status were compared. After a 4-week program, a survey was conducted; patients then completed a self-training program for 4 weeks, followed by a second survey.* Results*. During the initial 4 weeks, the EFT group experienced a significant decrease in the HB symptom scale, anger state, and paranoia ideation (*p* < 0.05). Over the entire 9-week interval, there were significant decreases in the HB symptom scale, anxiety state, anger state, anger trait, somatization, anxiety, hostility, and so on in EFT group (*p* < 0.05).* Conclusion*. The EFT group showed improved psychological symptoms and physical symptoms greater than those observed in the PMR group. EFT more effectively alleviated HB symptoms compared to PMR. EFT group showed better maintenance during self-training, suggesting good model of self-control treatment in HB patients.

## 1. Background

Hwabyung is a cultural syndrome in Korea and is well described in the Glossary of Culture-Bound Syndrome of Diagnostic and Statistical Manual of Mental Disorders, fourth edition (DSM-IV, Appendix 1) [1]. The term HB, which means “anger or fire” and “disease,” is known as a chronic anger syndrome. HB patients experience chronic suppressed anger and express unique symptoms including feelings of unfairness, subjective anger, external anger, heat sensation, pushing-up in the chest, dry mouth, and sighing and psychological symptoms such as feelings of unfairness and resentment [2]. HB is reported in 4.1% of the Korean general population and is more frequent in low-income middle-aged or older housewives [3]. As studies investigating HB treatment increase, nonpharmacologic treatment is receiving attention as a research target.

Progressive muscle relaxation (PMR) is a widely known relaxation technique introduced by Jacobson that aims to reduce residual tension and ultimately achieve a zero firing threshold through a progressive process of muscle relaxation. Jacobson's progressive muscle relaxation model was originally designed to relax approximately 30 muscles over an extended period of time, but a more widely used muscle relaxation technique developed by Bernstein is comparatively simple, relaxing just 16 muscles [4]. PMR can decrease physiological strain and heart acceleration through parasympathetic activation. This mechanism is the basis of evidence for its utility in heart disease prevention, cancer prevention, and rehabilitation [5]. Conventional studies have shown that PMR reduces various physical symptoms stemming from several psychological diseases. PMR has been shown to decrease anxiety and is especially effective at reducing insomnia, depression, and anxiety in elderly patients, as well as preventing both affective and behavioral disorders. PMR also reduces anxiety and psychological distress while improving subjective well-being in patients with schizophrenia [6]. It is also known to reduce heart rate and blood pressure [7]. PMR is currently utilized in HB patients, and studies have been conducted supporting its use [8].

The Emotional Freedom Technique (EFT) is a meridian-based psychological therapy that alleviates psychologic and psychosomatic conditions by applying tapping stimulations at certain meridian acupoints. This technique utilizes psychotherapy techniques such as assurance, while applying a tapping stimulation onto acupoints for meridian stimulation. Goodheart performed a meridian tapping therapy called emotional acupuncture; Thought Field Therapy (TFT) was later formalized by Callahan through numerous systematic studies investigating certain emotional problems such as fear and anxiety. In 1987, Carrigton created a technique of uniformly tapping 14 stimulation points called Acutap. Craig introduced a similar treatment in 1990, which became EFT in its present form. Those treatments were then generalized as Energy Psychotherapy and Meridian Tapping Therapy. In recent studies, EFT has been proven effective in alleviating symptoms such as headache [9], trauma [10], depression [11], phobia [12], insomnia [13], and anxiety [14]. Because these symptoms share certain similarities with those in HB, EFT is expected to be effective in HB patients. But because the time and frequency of the therapies can be changed variously, the standardization is needed to perform expected effect.

By accessing past memories and trauma, EFT lessens their impact on the patient in the present. Through this process, EFT is expected to reduce emotional trauma, a known HB trigger, in affected patients. PMR is thought to alleviate HB symptoms by fostering overall body relaxation. Therefore, PMR is expected to access trauma and lessen its influence in a manner different from EFT.

Accordingly, the present clinical trial investigates the effectiveness of EFT as a new treatment option for HB patients experiencing anger as a primary symptom and compares the efficacy to that of the conventional meditation technique of PMR.

## 2. Methods

### 2.1. Study Subjects and Trial Period

**Figure 1:**
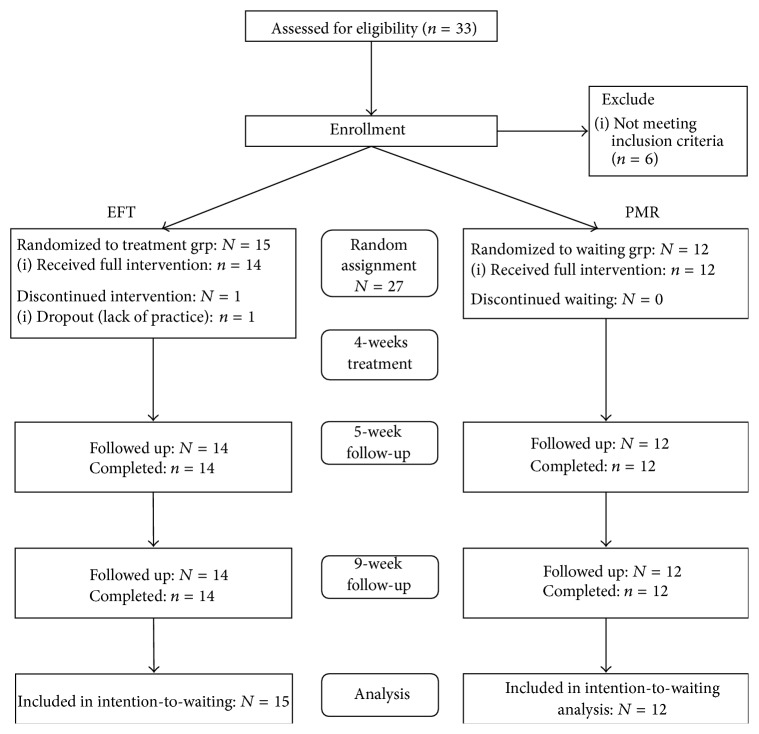
Schematic of the entire EFT and PMR program protocol.

Between November 2013 and February 2014, a total of 26 people participated in this clinical trial. The participants were patients diagnosed with HB verified using the HB SCID during a screening period. In total, 27 participants were eligible based on the HB SCID and were classified as subjects for the study. Participants were assigned to the EFT treatment group (*n* = 15) or the PMR treatment group (*n* = 12) by random allocation; 26 participants completed the study, and one subject in the EFT group dropped out in the middle of the study (Figure 1). The number of participants in the two groups differed because a random allocation was performed on two separate occasions.

This clinical trial was approved by the Institutional Review Board of Kyung Hee University Hospital at Gangdong, Korea (KHNMCOH 2013-01-012).

### 2.2. Program Protocol

Each subject group convened together for 1 hour each week to undergo EFT or PMR training. By providing education on HB, the basics concepts of meditation, and time for practice; the training program aimed to reduce the symptoms of HB.

The programs were performed over a 4-week period, and afterwards participants were instructed to complete homework using the distributed worksheets and self-training CD. These group programs are each summarized in Tables 1 and 2.

All groups were instructed to practice the program daily using the distributed text and mp3 files. All subjects recorded the number of daily practices and stressful events treated with the therapy. The EFT group (*n* = 15) and PMR group (*n* = 12) both performed the program.

*Group Treatment*. Both groups gathered weekly at the same time and location to receive group therapy and self-training goals.

The program in the EFT group followed the EFT Borrowing Benefit protocol [15] as follows:

 Goal setting and confirming the problem.
 Set-up.
 Sequence.
 The 9 Gamut sequence.
 Sequence.
 EFT reframing.

### 2.3. Symptom Assessment and Measurement Tools

To assess the condition of HB patients, the Hwabyung scale, STAI, STAXI, and SCL-90-R were utilized. Among these, the Hwabyung scale was used to assess HB-related symptoms and the severity of the psychological status. STAI and STAXI were employed to analyze the degree of anxiety and anger in the patients. Finally, the SCL-90-R was used to assess the overall psychological status of the participants.

#### 2.3.1. Hwabyung Scale

The Hwabyung scale is a tool used to measure the severity of Hwabyung-related symptoms in Hwabyung patients. It is the first self-reported survey measuring Hwabyung and was constructed by Kwon et al. [16]. All items on the Hwabyung scale as well as subscales of Hwabyung characteristics and Hwabyung symptoms have a relatively high degree of internal consistency, and the symptoms of Hwabyung differ significantly between Hwabyung groups and depression groups. The scale assesses symptoms of Hwabyung during primary screening up to 30 points [16].

#### 2.3.2. STAI

The STAI is used to measure the anxiety state of HB patients. Developed by Spielberger in 1964, STAI was designed as a subjective and easy-to-use self-reported scale that can measure both the anxiety state and trait simultaneously. Our study is based on the Korean version of the STAI [17].

#### 2.3.3. STAXI

The STAXI is a tool for assessing the status of anger. STAXI was developed by Spielberger in 1987 as a tool to measure components of anger and can be utilized to analyze normal and abnormal personality characteristics. A state of anger is defined as a diverse range of subjective feelings ranging from feeling hotly indignant to displaying outrageous anger. The trait of anger is defined as a tendency to interpret a wide range of conditions as anger provoking or frustrating. Eight questions each were also included focusing on the expression of rage, known as anger-out, the suppression of rage, called anger-in, and the attempted regulation of rage, called anger-control [18].

#### 2.3.4. The Symptom Checklist 90-R (SCL-90-R)

First developed and improved by Derogetis as a self-reported multidimensional clinical checklist examination, this test comprises nine symptom scales measuring somatization (SOM), obsessive-compulsive (OC), interpersonal sensitivity (IS), depression (DEP), anxiety (ANX), hostility (HOS), phobic anxiety (PHOB), paranoid ideation, and psychoticism (PSY) symptoms and three overall scales measuring the global severity index (GSI), positive symptom distress index (PSDI), and the positive symptom total (PST). This type of self-report is effective in detailing the subjective experiences of patients that an observer cannot detect and is therefore used as a primary screening tool in patients requiring intervention. Because this test requires patients to self-assess their own conditions, it enables patients to organize their symptoms and clinicians to readily assess the patient condition relatively quickly [19].

### 2.4. Statistical Analysis

Survey data were analyzed using SPSS (version 18) and Microsoft Excel (2010). Pre- and posttreatment comparisons within each group were performed using average value correspondence analysis, and an independent analysis was used for the pre- and posttreatment comparisons between the groups. Data from each scale were evaluated to determine whether they were nonparametric using the Shapiro-Wilk test. When the data were nonparametric (STAI anxiety state scale, STAXI anger state scale, OC of SCL-90-R, ANX, PHOB, HOS, PST, and PSDI scales), the Mann-Whitney *U* nonparametric test was performed to determine significance compared to baseline in each group. When a periodic variation was nonparametric (all of STAI, all of STAXI, SOM in SCL-90-R, HOS, PHOB, IS, DEP, ANX, PSY, GSI, and PST), the Wilcoxon matched pairs signed ranks test was performed to assess the significance of changes. For the T-score of the SCL-90-R, a nonparametric test, the Wilcoxon matched pairs signed ranks test, was performed for the SOM, IS, DEP, ANX, HOS, PHOB, PSY, GSI, and PST scales.

In the pre- and posttreatment comparisons within groups, changes occurred between the weeks 1 and 5 (termination of clinical education), and weeks 1 and 9 (termination of follow-up) were analyzed using the standard of verification for effectiveness. Statistical significance in each scale was designated as *p* < 0.05. An intention-to-treat analysis and per-protocol analysis was performed in both groups. The statistical analysis was performed by a blinded controlled specialist.

## 3. Results and Discussion

### 3.1. Results

#### 3.1.1. Differences within Each Group

There were no significant differences in the EFT and PMR groups compared to their respective baselines, except for the PHOB scale, which showed some difference. However, because the overall PHOB score was less than 70, which is the severe point of the T-score, it can be concluded that there was no significant clinical difference in this scale as well. There was no significant difference in the mean age between the EFT (53.53 ± 9.64 years) and PMR groups (59.00 ± 8.22 years, *p* = 0.131).

#### 3.1.2. Significant Differences in the Hwabyung Scale

There was a significant decrease in the Hwabyung symptom scale (*p* = 0.031) between baseline and week 5 in the EFT group. After concluding the program, there was a highly significant decrease in the Hwabyung symptom scale between baseline and week 9 (*p* = 0.001).

**Figure 2:**
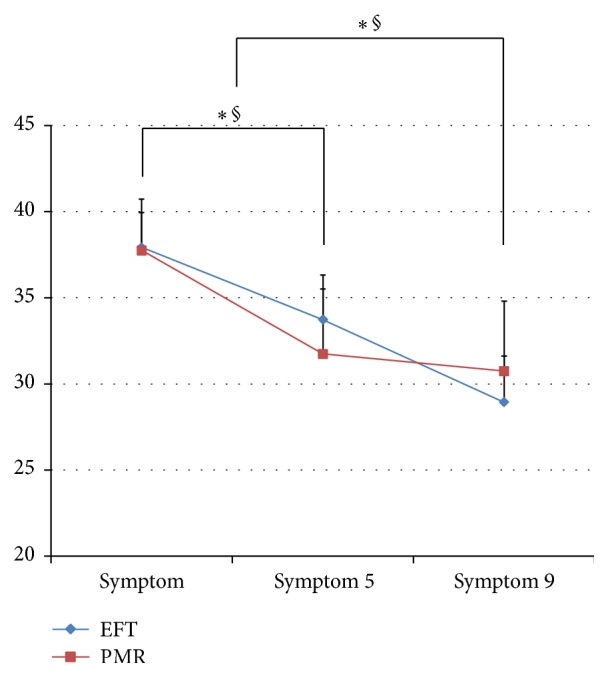
The Hwabyung symptom scale compared between the EFT and PMR groups over time. *∗* = *p* < 0.05 (EFT group); § = *p* < 0.05 (PMR group); symptom: baseline survey; symptom 5; 5th week survey; symptom 9: 9th week survey.

In the PMR group, there was a significant decrease in the Hwabyung symptom scale between baseline and week 5 (*p* = 0.049). Between baseline and week 9, there was also a significant decrease in the Hwabyung symptom scale in the PMR group (*p* = 0.041, Figure 2).

There were no significant differences between the two groups in differences in values before and after.

#### 3.1.3. Significant Differences in STAI

**Figure 3:**
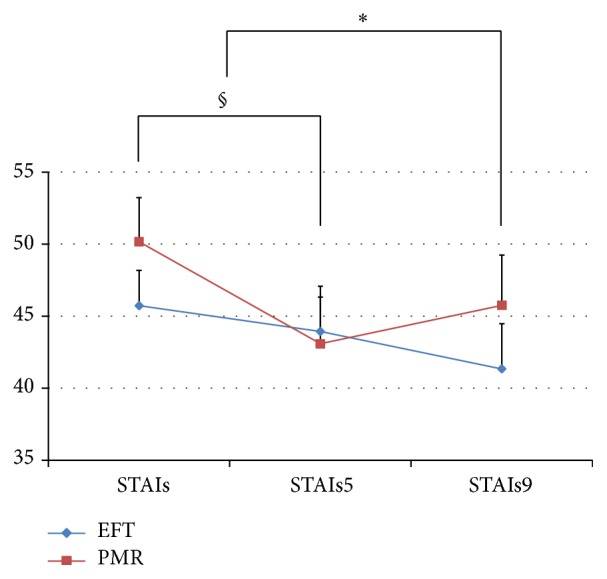
The STAI anxiety state scale compared between the EFT and PMR groups over time. *∗* = *p* < 0.05 (EFT group); § = *p* < 0.05 (PMR group); STAIs: baseline survey; STAIs5: 5th week survey; STAIs9: 9th week survey. The STAI anxiety state scale was analyzed using the Wilcoxon signed ranks test.

In the EFT group, there was no significant decrease in both the anxiety state and trait between baseline and week 5. After concluding the program, there was a significant decrease in the anxiety state between baseline and week 9 (*p* = 0.046, Figure 3).

In the PMR group, there was a significant decrease in both the anxiety state (*p* = 0.035) and trait (*p* = 0.032) between baseline and week 5. After completing the program, there was no significant change between baseline and week 9.

No significant pre-post change differences in STAI were found between the two groups.

#### 3.1.4. Significant Differences in STAXI

**Figure 4:**
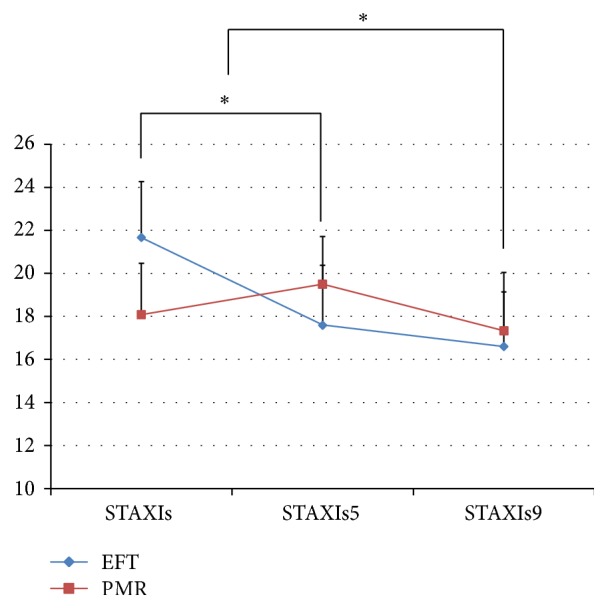
The STAXI anger state scale compared between the EFT and PMR groups over time. *∗* = *p* < 0.05 (EFT group); STAXIs: baseline survey; STAXIs5: 5th week survey; STAXIs9: 9th week survey.

**Figure 5:**
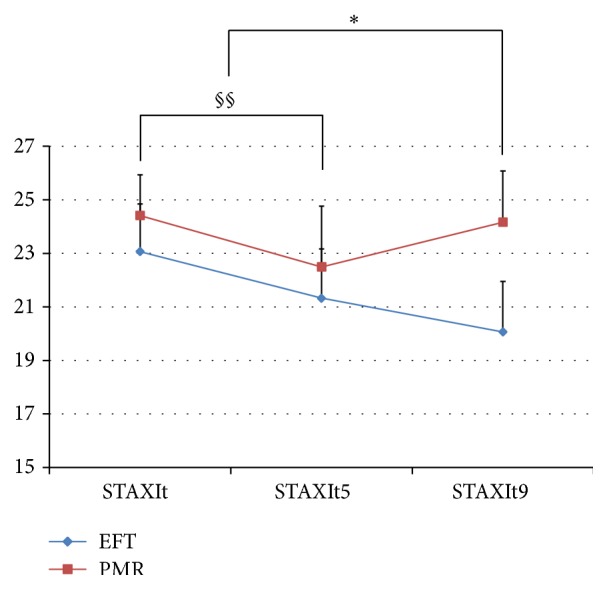
The STAXI anger trait scale compared between the EFT and PMR groups over time. *∗* = *p* < 0.05 (EFT group); § = *p* < 0.05 (PMR group); §§ = 0.05 < *p* < 0.1 (PMR group); STAXIt: baseline survey; STAXIt5: 5th week survey; STAXIt9: 9th week survey.

In the EFT group, there was a significant decrease in the anger state between baseline and week 5 (*p* = 0.021). After completing the program, there was a significant decrease in both the anger state (*p* = 0.006) and trait (*p* = 0.006, Figures 4 and 5).

In the PMR group, there was a decrease in the anger trait between baseline and week 5, while no significant difference was found between baseline and week 9 at the conclusion of the program.

No significant pre-post change differences in the anger trait in STAXI were found between the two groups. However, there was a significant difference in the anger state between the two groups between baseline and week 5 (*p* = 0.026) and baseline and week 9 (*p* = 0.047).

#### 3.1.5. Significant Differences in SCL-90-R

In the EFT group, there was a significant change in the paranoid ideation between baseline and week 5 (*p* = 0.012). After conclusion of the program, the EFT group showed significant decreases in somatization (*p* = 0.005), interpersonal sensitivity (*p* = 0.003), anxiety (*p* = 0.047), hostility (*p* = 0.003), paranoid ideation (*p* = 0.001), depression (*p* = 0.046), psychoticism (*p* = 0.047), the global severity index (*p* = 0.010), and the positive symptom total (*p* = 0.011) of SCL-90-R between baseline and week 9.

In the PMR group, there was a decrease in the hostility scale (*p* = 0.055) between baseline and week 5. After the conclusion of the program, the PMR group showed only a decreasing tendency in the hostility scale between baseline and week 9.

#### 3.1.6. T-Score Assessment of SCL-90-R

**Figure 6:**
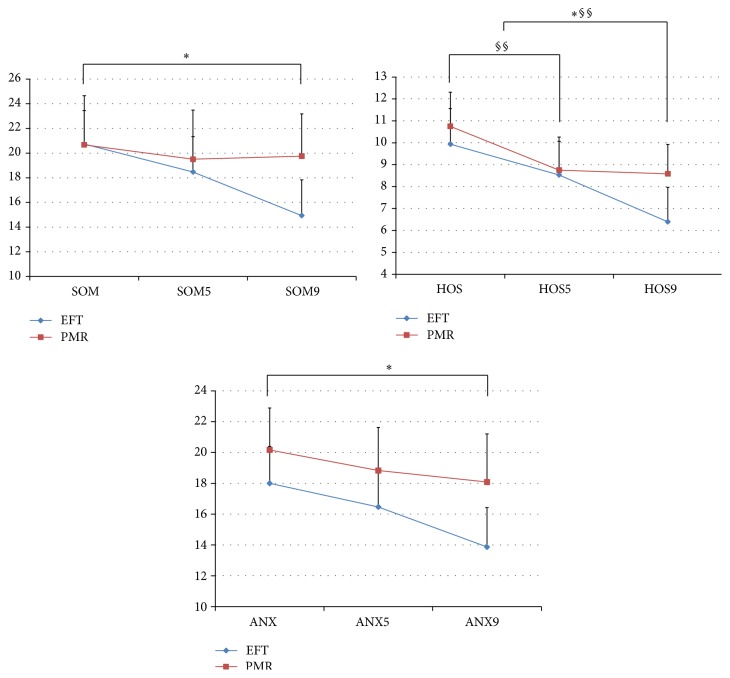
The SCL-90-R scales compared between the EFT and PMR groups over time. *∗* = *p* < 0.05 (EFT group); § = *p* < 0.05 (PMR group); §§ = 0.05 < *p* < 0.1 (PMR group); SOM: somatization baseline survey; SOM5: somatization 5th week survey; SOM9: somatization 9th week survey; HOS: hostility baseline scale; HOS5: hostility 5th week scale; HOS9: hostility 9th week scale; ANX: anxiety baseline scale; ANX5: anxiety 5th week scale; ANX9: anxiety 9th week scale.

In the EFT group, the SCL-90-R T-score only showed a significant decrease in the paranoid scale between baseline and week 5 (*p* = 0.016). However, between baseline and week 9, there was a significant decrease in somatization (*p* = 0.004), interpersonal sensitivity (*p* = −0.003), depression (*p* = 0.046), anxiety (*p* = 0.047), hostility (*p* = 0.003), paranoia (*p* = 0.001), psychoticism (*p* = 0.038), the global severity index (*p* = 0.014), and the positive symptom total (*p* = 0.010, Figure 6).

In the PMR group, the SCL-90-R T-score only showed a decreasing tendency in the hostility scale between baseline and week 5 (*p* = 0.068) and between baseline and week 9 (*p* = 0.074). The other scales showed no significant difference.

No significant pre-post change differences were observed in the T-score between the two groups.

### 3.2. Discussion

Hwabyung, an abbreviated form of Ulhwabyung, is a psychological syndrome where emotion such as anger explodes after being accumulated without appropriate resolution. Physical symptoms are characterized by chest pressure, a hot rash, and a feeling of constriction in the neck and xiphoid process. Feelings of resentment, anger, haan, and hard feelings characterize the psychological symptoms. The term is medically interpreted as somatization symptoms caused by prolonged exposure to stress. Because HB is characterized by emotional anger, it is often referred to as an anger syndrome. In Korean culture, especially, the concept of “haan” is occasionally linked to anger as an explanation of this disease.

Studies of Hwabyung have been performed to evaluate their psychological symptoms such as anxiety, anger, and depression. According to the reports, HB patients have difficulty in controlling anger caused by extreme anger suppression, with severe anxiety or depressive mood. These HB patients' psychological problems cause multiple symptoms including insomnia and heart palpitations. Various meditation techniques have been reported as effective in controlling these symptoms in numerous cases [20].

Several recent clinical trials have investigated the effects of EFT on symptoms of anxiety disorder including phobia, tensional headache, depression, anxiety, and insomnia [8–13], and a review reported that the technique was effective against emotional trauma such as PTSD [21]. Considering these therapeutic successes, EFT is expected to be more effective at controlling psychological problems rather than the physical symptoms.

As one of the most well-known relaxation therapies, PMR is widely used to alleviate pain in cardiac and other diseases. This behavioral treatment aims to relax muscles by repeatedly applying tension and relaxation, which reduces the physical symptoms of participants with relative ease. Randomized controlled trials have been conducted assessing the effect of PMR on adenomyosis patients [22] and breast cancer patients [23]. The trials reported significant improvements in the quality of life and alleviation of physical and psychological symptoms.

In this study, the PMR group was expected to show a greater decrease in physical symptoms compared to the EFT group. However, the EFT group improved more in physical symptoms, as well as the overall psychological statuses of anxiety and anger compared to the PMR group. Particularly on the Hwabyung symptom scale, EFT was confirmed to be a highly effective Hwabyung treatment in clinical conditions; the EFT group showed a mean score of less than 30 after 9 weeks of follow-up, which is considered marginal Hwabyung [24].

Participants in the EFT group were more effective at controlling Hwabyung symptoms, especially anger and anxiety, compared to the PMR group. This is clearly explained by the STAI, STAXI, and somatization, anxiety scales of the SCL-90-R. Therefore, the EFT program could be suggested as a method of controlling anger and anxiety symptoms in patients suffering from HB or other diseases. The significant decrease in the anger state scale of STAXI before and after treatment in both groups indicates that EFT is clearly effective for controlling a current state of anger.

In this study, we anticipated that patients in the EFT group would experience a feeling of group homogeneity during treatment. To utilize this effect in the analysis, the study was designed to maximize this synergistic effect. In order to match this study design, the PMR group was also subjected to group therapy. During the first 5 weeks of the study, patients were required to participate in the group sessions, and they then continued self-therapy without the group sessions during weeks 5–9. When the participants were analyzed through week 5, the EFT group showed significant improvements in more criteria than the PMR group did. In addition, a comparison between the baseline and final assessments showed that the EFT group experienced greater alleviation of symptoms than the PMR group did. While patients in the PMR group demonstrated a low level of symptom control after the group sessions, patients in the EFT group showed a continuous decrease in symptoms even during the self-training period without intervention. This clearly demonstrates that the EFT caused much improved self-training effect compared to PMR. The effect of group therapy was similar between both groups.

In addition, the effect of the group program in the EFT group continued to prevail during self-training after the fifth week. In other words, it means that EFT is more suitable at being performed on patients' own than PMR technique. It could be also inferred that EFT program itself was more effective considering the fact that homework distributed and CD shared an identical format. And because there were significant changes in several scales in week 9 compared to week 1, the program is expected to require a period longer than 8 weeks to show a full effect.

### 3.3. Limitations

The T-score was less than 70 in the SCL-90-R test, which means that the participants could actually be classified as normal emotional state. Further studies should be conducted in patients with severe psychological problems. In addition, the small number of participants limits the statistical significance of the findings; this limitation should be alleviated in further studies with big subjects. There is also an uncertainty and lack of control in programs conducted in the home after the group session is over.

## 4. Conclusions

1. The 4-week EFT program shows more effect to improve scales of psychological symptoms including anxiety and anger in HB patients than PMR program. The significant improvements in physical symptoms are also found in EFT group.
2. The EFT group showed greater decreases in HB symptoms compared to the PMR group. The EFT group also showed improvement or sustained improvement during the self-training period of weeks 5–9. This demonstrates that EFT is an effective self-control treatment for HB.
3. PMR was proven effective on the Hwabyung scale symptoms. This is consistent with results from conventional studies showing that PMR is effective in alleviating physical symptoms.

## Figures and Tables

**Table 1:** The Emotional Freedom Technique (EFT) program performed on the EFT group (EFT Borrowing Benefit).

Weeks	Program contents
1st week	(i) Education on Hwabyung (ii) Introduction of basic EFT (iii) Goal setting and confirming the problem (iv) Set-up (v) The sequence (vi) The 9 Gamut sequence (vii) Treat present symptom (viii) Share experiences with Hwabyung (ix) Distribute worksheets, self-training CD, and homework assignment

2nd week	(i) Review week 1 program (ii) Share experiences from the previous week (iii) Treat past trauma (iv) Inquire about the person and the events that caused Hwabyung (v) Education on EFT reframing (vi) Distribute worksheets, self-training CD, and homework assignment

3rd week	(i) Review weeks 1 and 2 programs (ii) Share experiences from the previous week (iii) Treat distorted self-image (iv) Distribute worksheets, self-training CD, and homework assignment

4th week	(i) Review weeks 1–3 programs (ii) Share experiences from the previous week (iii) Treat the doubtful future (iv) Distribute work sheets, self-training CD, and homework assignment

**Table 2:** Progressive muscle relaxation (PMR) performed on the PMR group [15].

Weeks	Program contents
1st week	(i) Education on Hwabyung (ii) Introduction of PMR and meridian muscle points (iii) Practice PMR on the upper body (iv) Share experiences with Hwabyung (v) Distribute worksheets, self-training CD, and homework assignment

2nd week	(i) Review week 1 program (ii) Share experiences from the previous week (iii) Practice PMR of face and chest (iv) Distribute worksheets, self-training CD, and homework assignment

3rd week	(i) Review weeks 1 and 2 programs (ii) Share experiences from the previous week (iii) Practice PMR of the lower body (iv) Application in stressful circumstances (v) Distribute worksheets, self-training CD, and homework assignment

4th week	(i) Review weeks 1–3 programs and all practices of the entire body (ii) Share experiences from the previous week (iii) Application in stressful circumstances (iv) Distribute worksheets, self-training CD, and homework assignment
